# Automated serial extraction of DNA and RNA from biobanked tissue specimens

**DOI:** 10.1186/1472-6750-13-66

**Published:** 2013-08-19

**Authors:** Lucy Mathot, Monica Wallin, Tobias Sjöblom

**Affiliations:** 1 Science for Life Laboratory, Department of Immunology, Genetics and Pathology, Uppsala University, SE-751 85, Uppsala, Sweden

**Keywords:** Nucleic acid extraction, Cancer, Open automation, Sample preparation, Tissue biobanking

## Abstract

**Background:**

With increasing biobanking of biological samples, methods for large scale extraction of nucleic acids are in demand. The lack of such techniques designed for extraction from tissues results in a bottleneck in downstream genetic analyses, particularly in the field of cancer research. We have developed an automated procedure for tissue homogenization and extraction of DNA and RNA into separate fractions from the same frozen tissue specimen. A purpose developed magnetic bead based technology to serially extract both DNA and RNA from tissues was automated on a Tecan Freedom Evo robotic workstation.

**Results:**

864 fresh-frozen human normal and tumor tissue samples from breast and colon were serially extracted in batches of 96 samples. Yields and quality of DNA and RNA were determined. The DNA was evaluated in several downstream analyses, and the stability of RNA was determined after 9 months of storage. The extracted DNA performed consistently well in processes including PCR-based STR analysis, HaloPlex selection and deep sequencing on an Illumina platform, and gene copy number analysis using microarrays. The RNA has performed well in RT-PCR analyses and maintains integrity upon storage.

**Conclusions:**

The technology described here enables the processing of many tissue samples simultaneously with a high quality product and a time and cost reduction for the user. This reduces the sample preparation bottleneck in cancer research. The open automation format also enables integration with upstream and downstream devices for automated sample quantitation or storage.

## Background

Efficient methods for biomolecule extractions from many tissue samples simultaneously are key components of the future workflow in molecular profiling of tumors, in particular in cancer biobanking, research and diagnostics. A major limitation of current automated procedures is that they are not developed and validated for the extraction of nucleic acids from tissue samples. Tissue samples differ from blood samples in that they are heterogeneous and may vary in composition from samples with high fat content and low cell number, such as normal breast, to very fibrous samples such as muscle and cell dense samples like spleen. It has therefore not been common practice to use a universal extraction process for all tissue types; different tissue types have often been processed manually with different extraction kits or with titrated input amounts, which is expensive and time consuming [1]. The quality of biomolecules extracted from tissues is variable, and depends on many factors, including time from removal from the patient to freezing or fixation, sectioning methods employed by the pathology department and storage following sectioning [2,3]. When possible, it is preferable to use fresh frozen tissues for extracting RNA of high integrity and long strands of genomic DNA [4].

Here, we develop and validate an automated extraction process using magnetic silica bead technology suitable for the serial extraction of DNA and RNA from many different types of solid tissues using a minimal number of reagents [5]. This process was designed to use fresh frozen tissue as starting material to obtain the highest quality DNA and RNA for downstream genomic and transcriptomic analyses, and therefore extracts both DNA and RNA from the same cells of the same tumor tissue [6]. To demonstrate performance and scalability, we processed 864 solid tissue specimens and assessed the extracted materials in several downstream applications, including PCR-based STR analysis, deep sequencing on an Illumina platform following a HaloPlex selection, and gene copy number studies using microarrays.

## Methods

### Tissue collection and preparation

864 tumor-normal paired colorectal and breast frozen tissue samples (288 colorectal, 576 breast) as well as 30 samples of liver, prostate, tonsil, colon, breast, thymus, kidney, skin, uterus and lung were obtained from the frozen tissue collection at the Department of Pathology, Academic Hospital Uppsala. This study was approved by the Regional Ethical Review Board of Uppsala (2007/116) and written consent was obtained from participants. The tissues were embedded in OCT and stored at −80°C to maintain biomolecular integrity [1]. The breast and colon tumor sections contained a minimum of 50% and 40% tumor cells, respectively. The blocks were sectioned and 2 or 3 10 μm sections per specimen were collected in 2D barcoded tubes in tube racks of 96 (Micronic Roborack-96, art. no. MPW51016BC3).

### Automated serial extraction of nucleic acids

We recently described a novel process for serial DNA and RNA extraction employing silica beads with differential nucleic acid binding affinities [5]. This extraction procedure was automated on a liquid handling workstation (Tecan Evo 150 MCA LiHa RoMa) equipped with wash stations for 96 and 8 tips, respectively, a twin-block heater with two different constant temperatures (EchoTherm IC22, Torrey Pines Scientific), and readers for 1D plate barcodes (Symbol MS954) and 2D tube barcodes (Ziath), respectively. Briefly, nine 96-well plates of approximately 25 mg fresh frozen tissue from 864 patient-matched tumor and normal tissues (288 colorectal, 576 breast) were collected as described. Unless otherwise stated, all liquid transfers were performed with a 200 μL fixed tip block (Tecan). At the start of each run, all reagents along with one SBS format tube rack with 96 samples were loaded on the robotic workstation and uncapped. The lysis buffer was dispensed using an 8-channel LiHa pipetting head. After addition of chaotropic lysis buffer, the samples were incubated for 15 min at 58°C, followed by incubation with DNA binding beads for 15 min. All liquid handling after the initial dispensing of lysis buffer to the tissue samples was performed using a 96-tip MCA pipetting head with tip washes between each process step.

The DNA binding beads were captured using a magnetic plate (V&P Scientific) and the supernatant transferred into a new vessel for RNA capture. Meanwhile, the DNA selective beads were washed three times in wash buffer and bound DNA eluted in TE buffer. After 15 min binding, the RNA binding beads were retrieved using a magnetic plate and washed first in DNase (ThermoScientific) containing wash buffer and thereafter washed and eluted in the same buffer composition as used for the DNA extraction [5]. The final DNA and RNA products were transferred to a 96 well Roborack barcoded storage plate (Micronic, Article No MPW51016BC3). The worktable layout is shown in Additional file 1: Figure S1.

### Quality control of extracted DNA and RNA

The DNA yields from the tissue samples were assessed by measurements using a High Sensitivity dsDNA kit on a Qubit® instrument (Invitrogen). The purity of the DNA was assessed by spectrophotometry (OD 260:280 ratio) using a Nanodrop instrument (Thermo Scientific). The integrity of DNA was assessed by separation in a 0.7% agarose gel (Sigma Aldrich) and staining with SYBR Safe (Invitrogen). The integrity of selected RNA samples was assessed using an RNA 6000 Pico Assay on an Agilent 2100 Bioanalyzer instrument (Agilent). The samples were diluted 1:10 or 1:20 in RNase free water and denatured for 2 min at 70°C before separation. The 28S/18S ribosomal RNA ratio and RNA integrity (RIN) scores were computed using the Agilent Technologies 2100 Expert software package.

### Performance evaluation in genomic analyses

The extracted DNA was used in several downstream applications including PCR-based STR analysis, deep sequencing on an Illumina platform following a HaloPlex selection (Agilent), Sanger sequencing and gene copy number analyses.

PCR-based STR analysis was performed on 238 colorectal DNA samples (119 tumor/normal pairs). Briefly, 24 STR markers in regions showing loss of heterozygosity in cancer were amplified using a touchdown PCR protocol. PCR amplification was carried out using 2.5 ng of genomic DNA as template. The primers were each conjugated to one of the 3 fluorophores FAM, NED, or VIC (Sigma-Aldrich, Applied Biosystems). PCR was performed in 10 μL reactions containing 1 × PCR buffer (67 mM Tris–HCl, pH 8.8, 6.7 mM MgCl_2_, 16.6 mM NH_4_SO_4_, 10 mM 2-mercaptoethanol), 1 mM dNTPs, 1 μM forward and 1 μM reverse primers, 6% DMSO, 2 mM ATP, 0.25 U Platinum *Taq* (Invitrogen) and 2.5 ng DNA. Reactions were carried out in 96-well ABI 2720 thermocyclers using a touchdown PCR protocol (1 cycle of 96°C for 2 min; 3 cycles of 96°C for 10 sec, 64°C for 10 sec, 70°C for 30 sec; 3 cycles of 96°C for 10 sec, 61°C for 10 sec, 70°C for 30 sec; 3 cycles of 96°C for10 sec, 58°C for 10 sec, 70°C for 30 sec; 41 cycles of 96°C for 10 sec, 57°C for 10 sec, 70°C for 30 sec; 1 cycle of 70°C for 5 min). Fluorescently labeled PCR products were analyzed by fragment analysis in a capillary sequencing instrument (ABI PRISM 3730xl) using ROX500 (Applied Biosystems) as size standard followed by allele identification using GeneMapper Software v4.1 (Applied Biosystems).

Haloplex target enrichment for second-generation sequencing (Agilent) of 540 genes potentially implicated in colorectal cancer was performed on 400–800 ng DNA from 192 colorectal samples (96 tumor/normal pairs) according to the manufacturer’s instructions [7]. The enriched and barcoded targets were then deep sequenced on an Illumina next generation sequencing platform (Illumina) [8]. Sanger sequencing of the PCR products amplified for mutation validation was carried out by an initial touchdown PCR protocol as described above, using the 192 samples previously deep sequenced on an Illumina platform as DNA template. Following this, 18 μL reactions were prepared containing 20 ng PCR product template and 4 pmol M13 primer (Biomers). The sequence reactions were delineated at Uppsala Genome Center on an ABI PRISM 3730xl sequencing apparatus (Applied Biosystems).

Gene copy number analyses of 70 of the colon cancer samples were performed using Genome Wide SNP6 microarrays (Affymetrix), according to the manufacturer’s instructions.

## Results

### Automated biomolecule extraction from tissues

**Figure 1:**
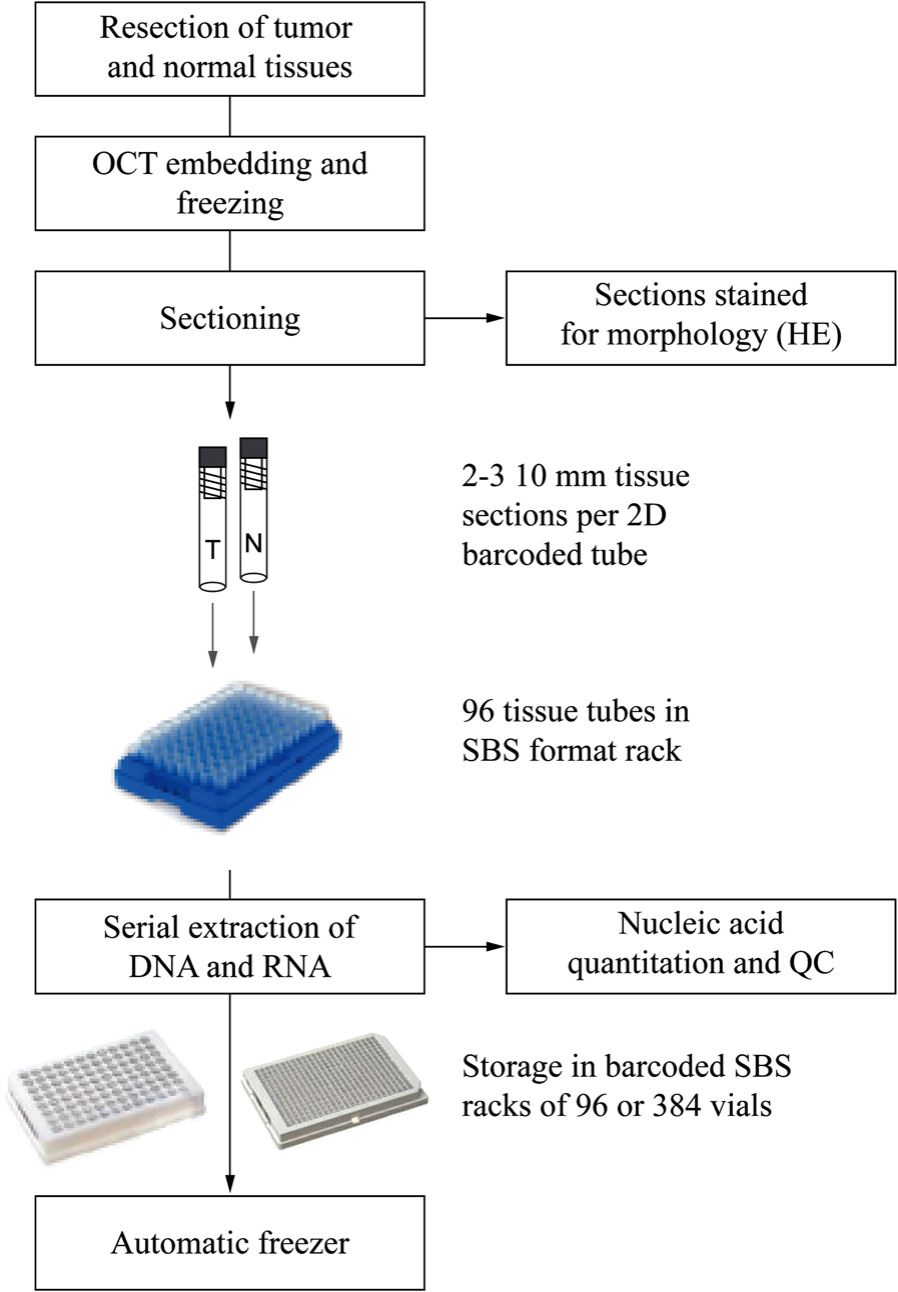
**A workflow for serial extraction of DNA and RNA in tissue biobanks.** Tumor and patient-matched normal biopsies and surgical specimens are embedded in OCT compound and frozen. Next, tissue sections are sectioned and stained for inspection by a pathologist and subsequent sections put in barcoded tubes in a tube rack for extraction. When a full rack of specimens has been generated, DNA and RNA molecules are serially extracted using the process presented in this work. The resulting nucleic acid fractions are quantitated and packaged in tube racks of sortable 96- or 384-well tubes for storage in automated freezers.

The serial DNA/RNA extraction method described in [5] was successfully implemented in a fully automated fashion on a Tecan Freedom Evo workstation for parallel extraction of 96 samples or 1–96 samples using 8-channel liquid handling. A tissue biobank workflow for integration of the extraction process is outlined in Figure 1. By omitting the grinding and shaking step of the original procedure, the number of transfers and the amount of hardware required in the robotic platform were reduced while maintaining yields and biomolecule integrity (data not shown). The total run time for extraction of 96 tissue samples was 1 h 40 min or 3 h 20 min when DNA alone or DNA and RNA, respectively, was recovered. The dropout rate (no DNA in eluate) for the initial 576 samples was ~ 2%, and the dropouts were likely caused by tissue clogging of the narrow bore fixed pipette tips. The remaining 288 samples were extracted using a wider bore 96 tip block (Tecan, art. no. 10290619), and there were no sample dropouts in this set.

### Serial recovery of high quality DNA and RNA from tissue specimens

**Figure 2:**
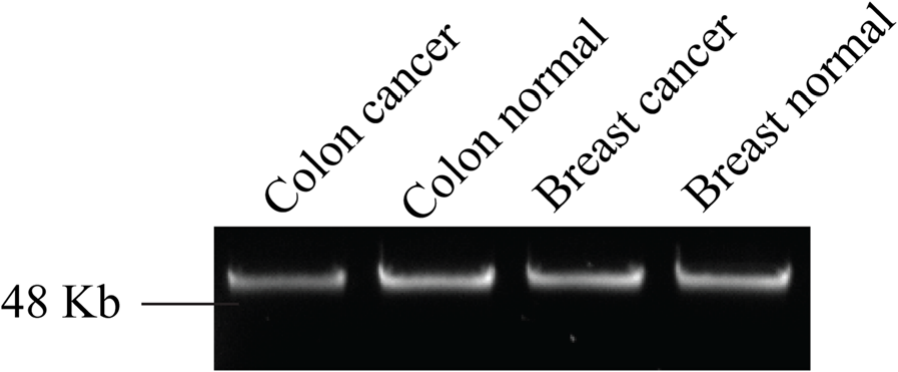
**The extracted DNA is of high molecular weight and integrity.** Electrophoretic separation of genomic DNA from colorectal and breast tissues on a 1% agarose gel.

The mean DNA yield of all samples was 3.2 μg (SD = 0.08, n = 576) as measured by a Nanodrop instrument and 0.9 μg (SD = 0.17) as measured by Qubit. The mean 260:280 ratio, used as a measure of protein contamination, was 1.66. The median 260:230 ratio, used as a secondary measurement of purity and indicating the presence of salts and other contaminants, was 1.57. The integrity of four randomly selected DNA samples is shown in Figure 2, where long strands of gDNA can be seen on a 1% agarose gel stained with SYBR Safe. Yields and purity (assessed spectrophometrically) of breast and colon DNA samples are presented in Table 1.

**Table 1:** Yield and purity of DNA extracted from 552 colon and breast samples

**Sample type**	**Mean DNA yield (μg)**	**Standard deviation**	**Median OD 260:280**	**Median OD 260:230**
Colon (n = 276)	3.15	0.14	1.73	1.72
Breast (n = 276)	3.25	0.87	1.49	1.09

### Storage stability of extracted RNA

**Figure 3:**
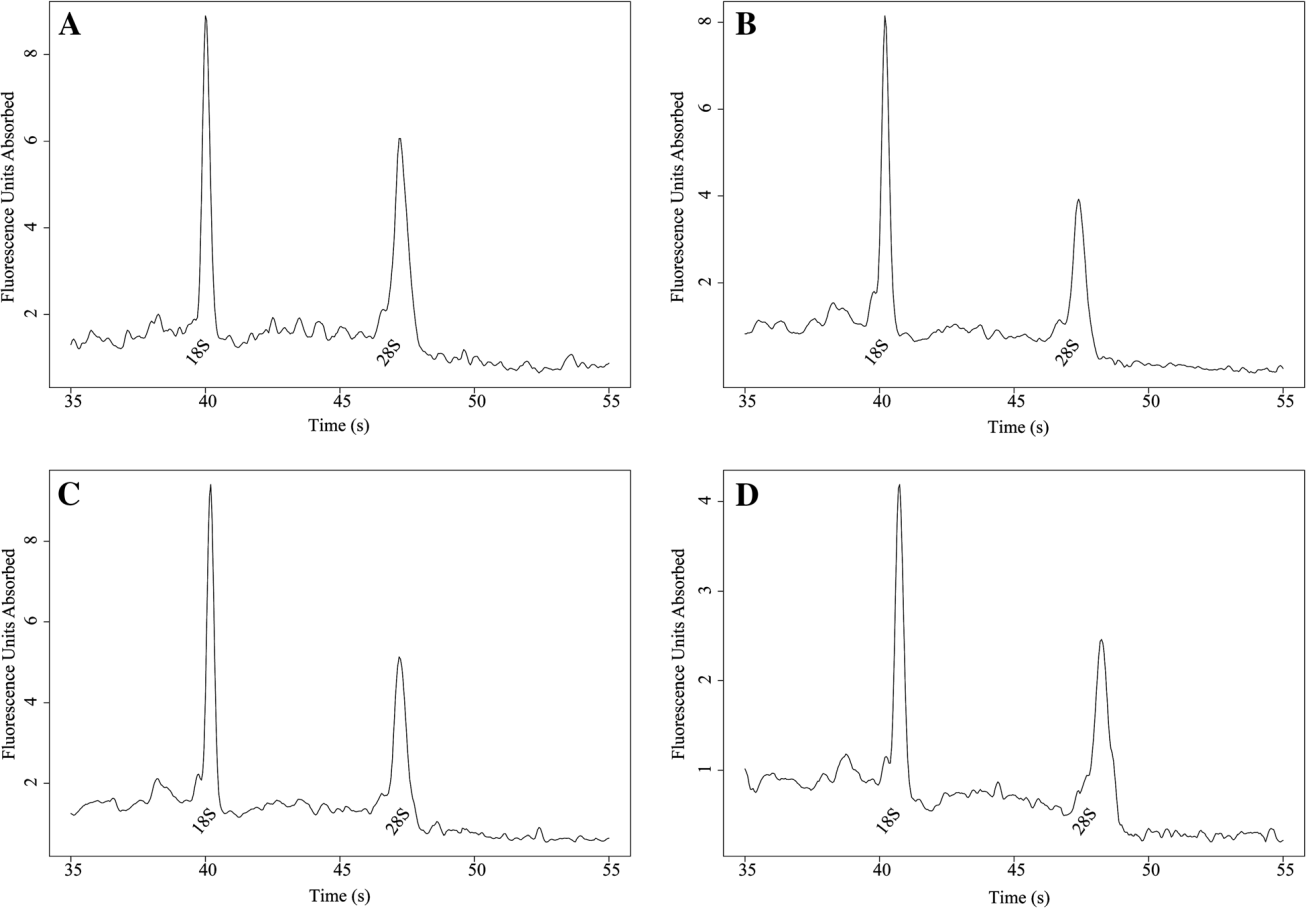
**The extracted RNA maintains integrity upon storage.** Electrophoretic separation of RNA on a Bioanalyzer using an RNA 6000 Pico chip, illustrating the integrity of diluted RNA at four time points measured every 3 months after initial extraction. **A**. represents integrity at time zero (1:10), **B**. at 3 months (1:10), **C**. at 6 months (1:10) and **D**. at 9 months (1:20).

The integrity of eleven randomly chosen RNA samples were measured using an RNA 6000 Pico assay. The RNA quality was found to be acceptable for use in most downstream processes, with an average RIN value across 11 samples of 6.85 at time zero. To assess storage stability, the samples were stored at −70°C and assessed at 3 months (average RIN = 6.69), 6 months (average RIN = 6.86) and 9 months (average RIN = 6.45), demonstrating stability under these conditions even with several freeze-thaw cycles. Peak profiles from each time point are shown in Figure 3. We have previously demonstrated the successful use of RNA from this extraction chemistry in RT-PCR [5].

### Conventional and next-generation sequencing analyses of genomic DNA

The extracted DNA was first used in PCR-based short tandem repeat (STR) analysis to compare genomic loci between tumor/normal matched samples to ensure that they were correctly paired. This allowed the detection of loss of heterozygosity in chromosomally unstable samples, as well as revealing that two of the paired samples were mismatched (Additional file 2: Figure S2).

**Figure 4:**
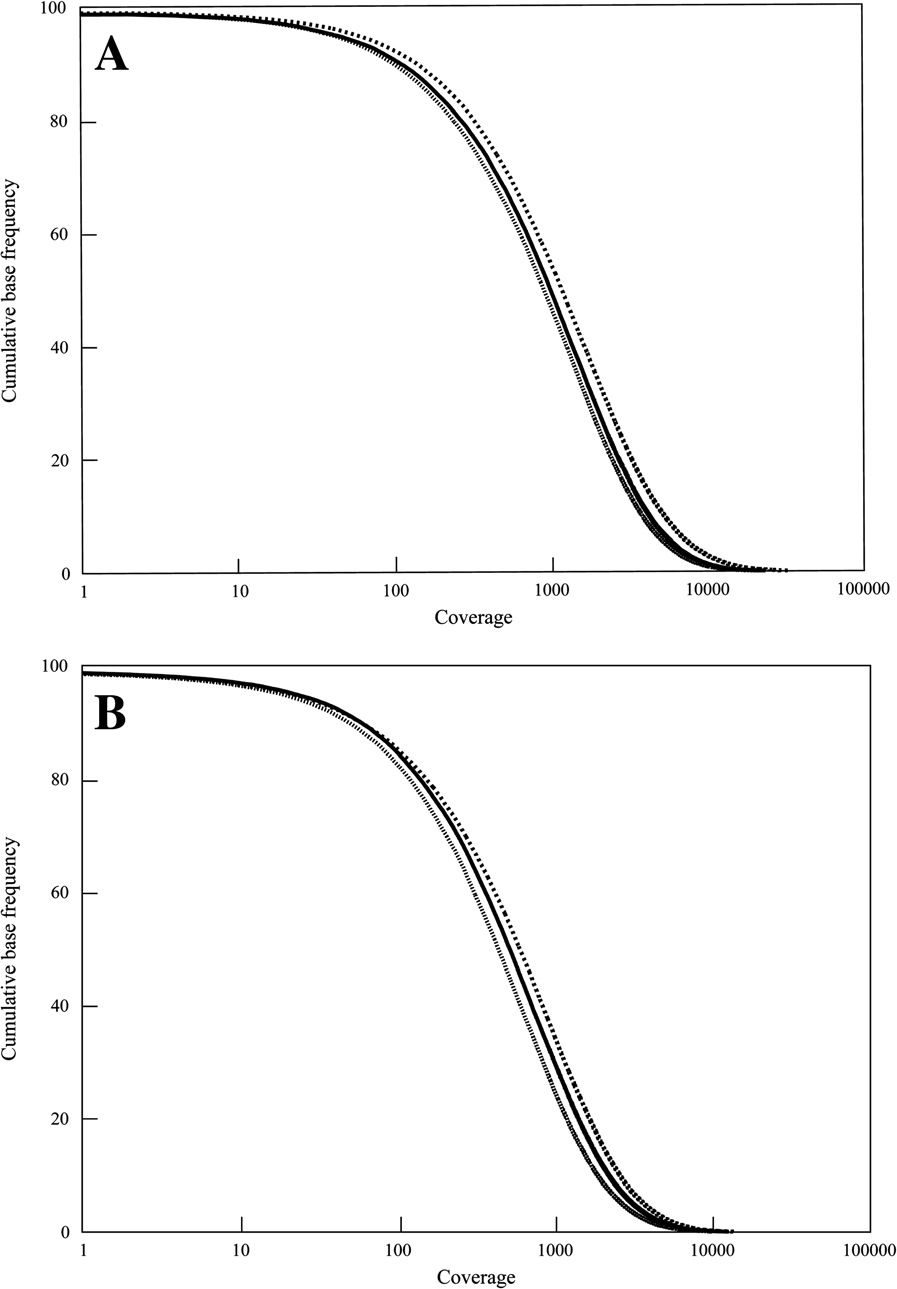
**The extracted DNA is suitable for target enrichment and sequencing by next generation technologies. A**. Sequence coverage of the median (solid line), first quartile (closely dotted line) and third quartile (dotted line) tumor samples (n = 96), while **B**. Sequence coverage of the median (solid line), first quartile (closely dotted line) and third quartile (dotted line) normal samples (n = 96).

Next, 400 – 800 ng DNA (as measured by Qubit) from 96 pairs of matched tumor and normal colorectal tissue (192 samples in total) was used in a Haloplex target enrichment and barcoding protocol (Agilent) and sequenced on an Illumina platform to identify driver mutations and key pathways involved in colorectal cancer (CrC) (Mathot *et al.*, in progress). Plots of the fraction of regions of interest covered by at least a certain read depth, illustrating the quality of the data produced by the NGS technology for both the normal and tumor samples are shown in Figure 4. The mean read depth was approximately 500 for the normal samples and 1000 for the tumor samples.

Identification of genomic aberrations using Genome Wide SNP6 arrays (Affymetrix) was also carried out on 70 of the CrC samples. The mean sample quality (QC) value as calculated by Nexus Copy Number™ software from Biodiscovery (measuring the probe to probe variance) was 0.19 (SD = 0.05). A QC value in the range 0.15 - 0.20 is considered high quality, with higher quality samples approaching 0.2.

## Discussion and conclusions

Sample acquisition and preparation is becoming the most time consuming step in large scale genomic analyses of solid tumors. We have therefore designed, implemented and validated an automated method for the serial extraction of DNA and RNA molecules from tissues of various types, with a particular view to using this method in cancer genomic and transcriptomic studies. The automation solution proposed here enables a high-throughput, cost-effective preparation of samples with minimal hands-on time.

Tissue extraction presents a distinct set of problems not applicable to blood or body fluids. Most extraction methods, in particular with regard to RNA, require a titration of input material to determine the optimal input, to avoid overloading the binding capacity [1]. Here we describe a method that can extract uniformly from a similar amount (approximately 25 mg each) of a variety of input tissue types. The technique produces, on a large scale, nucleic acids of high quality suitable for many downstream processes. The process is suited to a wide variety of tissue types, and has successfully been used to extract more than ten different tumor and normal tissues, including those of the liver, prostate, tonsil, colon, breast, thymus, kidney, skin, uterus and lung (data not shown). The extraction yield and purity is identical with manual extraction using the same chemistry [5]. The method presented here performs well when compared to other established extraction techniques, despite the omission of extensive tissue homogenization steps. (Using a standard phenol-chloroform extraction technique with an overnight Proteinase K digestion on 25 mg of colon tissue resulted in a DNA yield of 0.8 – 1.2 μg as measured on the Nanodrop).

The quality of the extracted biomolecules was validated by several different methods, commonly employed in cancer genetics. The extracted DNA was of high molecular weight with no apparent fragmentation, which is essential in whole genome sequencing approaches. The OD 260:280 ratio (measured by Nanodrop), frequently used as an indication of protein contamination, was within a range suitable for DNA analysis [9]. The OD 260:230 ratio (Nanodrop), used as a measure of the purity of DNA, is slightly low, likely due to the absorbance of residual guanidine at 230 nm [10]. This is inherent to methods using chaotropic lysis buffers. However, the performance of extracted DNA in any of the downstream applications tested was not affected, even when using microarrays known to be sensitive to low OD 260:230 ratios [11]. The concentration of double stranded DNA measured by the fluorometric Qubit method, proved to be a more useful measurement of amplifiable DNA, and compared well with real time PCR amplification of LINE1 elements (data not shown) [12]. The differences between Qubit and Nanodrop measurements may be explained by the fact that the Nanodrop instrument measures both single and double stranded DNA, as well as single nucleotides, giving an overall higher DNA yield than the Qubit method [10]. The performance and uniformity in next-generation sequencing applications was validated by targeted enrichment and sequencing of the exons of 540 genes in 192 tumor and normal colorectal tissue samples (Figure 4). RIN values for RNA extracted from tissue can be variable and depend greatly on the sectioning process and storage conditions prior to extraction [13]. The extracted RNA had RIN values near 7, which is suitable for many techniques used to study RNA, e.g. cDNA generation by RT-PCR and microarray analyses. In fact, values above 5.5 are sufficient for most applications [13]. We noted during development of the process that prior recovery of DNA facilitates RNA recovery, thereby contributing to increased quality of the RNA obtained [5].

Future developments include adapting the method to extraction of nucleic acids from formalin-fixed, paraffin-embedded (FFPE) tissues. In addition, in light of the finding that sample mix up is a common problem in cancer genetic studies (here illustrated by two mismatched pairs out of 96), we have recognized the need for robust and scalable identification methods. An automatable genotyping method for possible incorporation to the process, targeting insertion and deletion polymorphisms has also been developed [14]. Taken together, we have developed a walk-away automation solution to process fresh-frozen tissue specimens to high quality biomolecules ready for use in cancer research and diagnostics. This novel technology enables the simultaneous processing of many different types of tissue samples in the pathology biobank workflow with a time and cost reduction for the user.

## Abbreviations

PCR: Polymerase chain reaction; STR: Short tandem repeat; RT-PCR: Reverse transcriptase polymerase chain reaction; gDNA: Genomic DNA; OCT: Optimal cutting temperature; TE: Tris EDTA; OD: Optical density; SD: Standard deviation; RIN: RNA integrity number; NGS: Next generation sequencing; CrC: Colorectal cancer; QC: Quality control; LINE: Long interspersed nuclear elements; FFPE: Formalin-fixed, paraffin-embedded.

## Competing interests

The authors are shareholders in ExScale Biospecimen Solutions AB, which commercializes the technology for scalable extraction described here.

## Authors’ contributions

ML and LM performed the automated extractions and sample measurements. LM used the extracted material in downstream analyses and drafted the manuscript. TS conceived of the study, and participated in its design and coordination and helped to draft the manuscript. All authors read and approved the final manuscript.

## Supplementary Material

Additional file 1: Figure S1 The worktable layout on the Tecan Freedom Evo robotic workstation. Click here for file

Additional file 2: Figure S2 DNA extracted can be incorporated into PCR-based downstream analysis. Capillary electrophoretograms from the STR analysis of matched and unmatched tumor/normal pairs. A. shows a matched tumor/normal pair, B. shows an unmatched tumor/normal pair. Click here for file
