# Supplementary figures and images for: Automated serial extraction of DNA and RNA from biobanked tissue specimens
Source: BMC Biotechnol. 2013 Aug 19;13:66. doi: 10.1186/1472-6750-13-66 (PMC3751724; doi:10.1186/1472-6750-13-66)

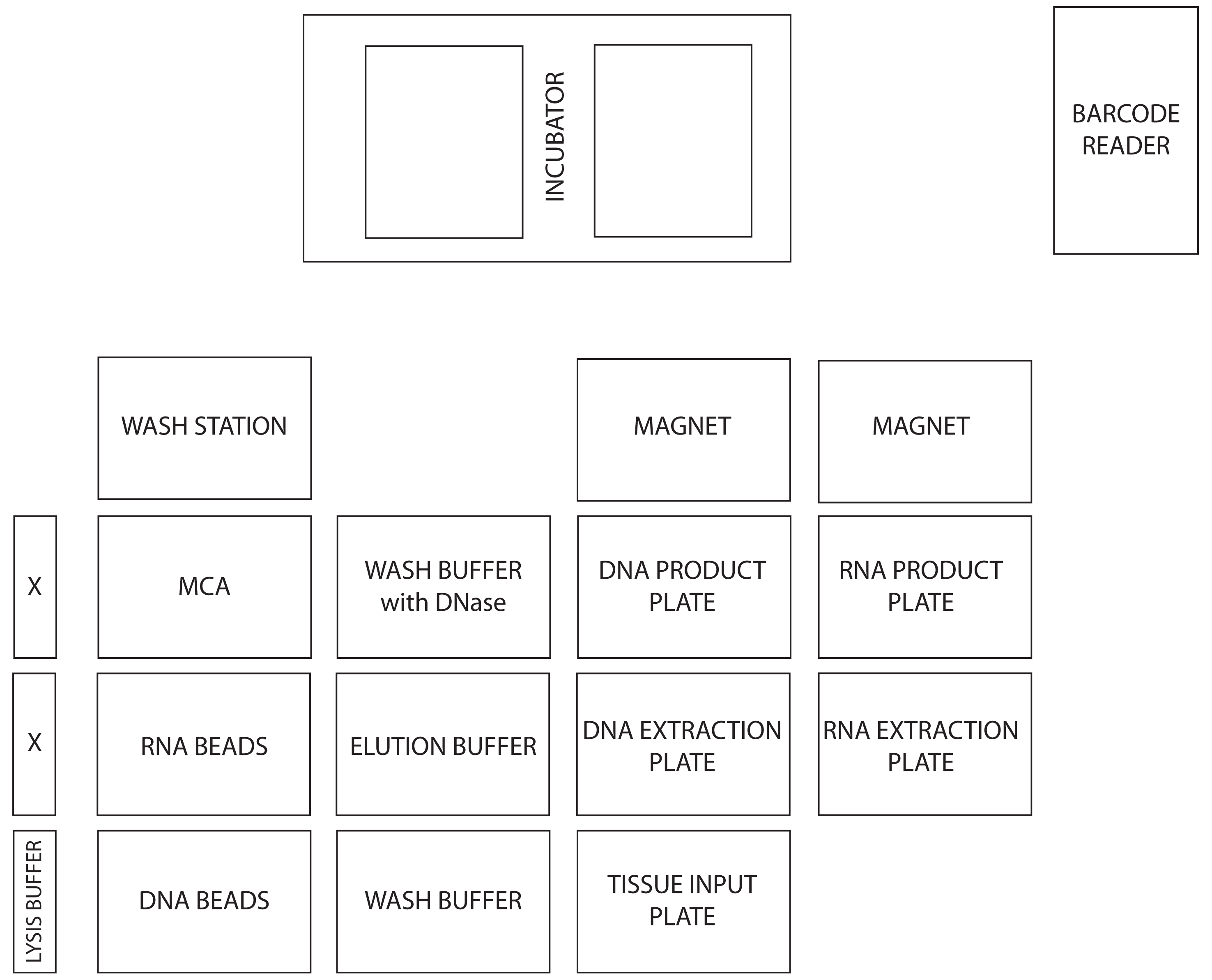

Supplement: Additional file 1: Figure S1 — The worktable layout on the Tecan Freedom Evo robotic workstation. [file 1472-6750-13-66-S1.tiff]

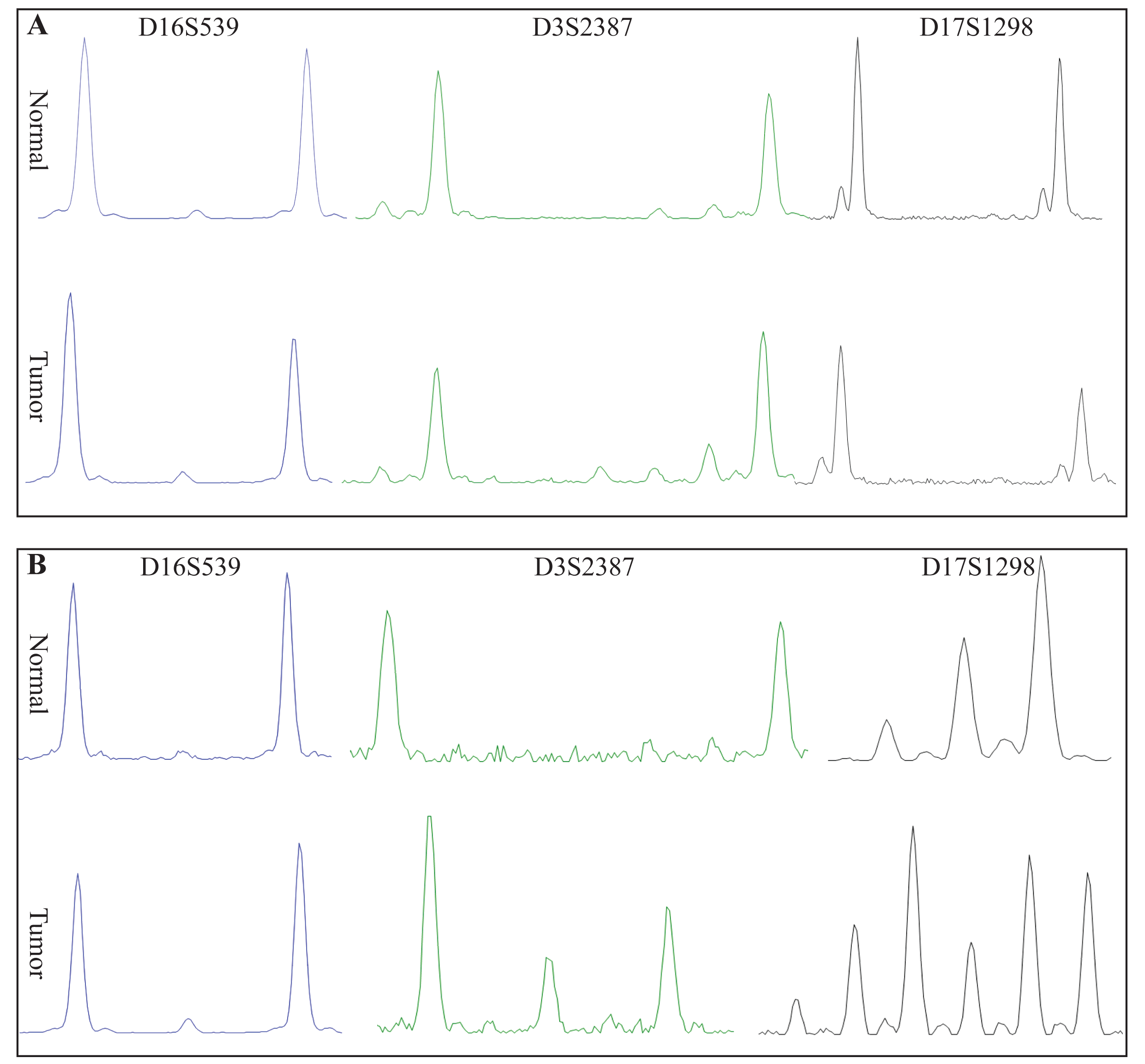

Supplement: Additional file 2: Figure S2 — DNA extracted can be incorporated into PCR-based downstream analysis. Capillary electrophoretograms from the STR analysis of matched and unmatched tumor/normal pairs. A. shows a matched tumor/normal pair, B. shows an unmatched tumor/normal pair. [file 1472-6750-13-66-S2.tiff]
